# Feasibility and Safety of Concomitant Laparoscopic Cholecystectomy With Open-Heart Surgery: A Systematic Review and Our Early Clinical Experience

**DOI:** 10.7759/cureus.52844

**Published:** 2024-01-24

**Authors:** Shresth Manglik, Camelia Pal, Urmila Basu, Lalit Kapoor, Pradeep Narayan, Sanjay K Dubey

**Affiliations:** 1 Department of General Surgery and Minimally Invasive Surgery, Narayana Hospital - Rabindranath Tagore International Institute of Cardiac Sciences, Kolkata, IND; 2 Department of Cardiothoracic and Vascular Surgery, Narayana Hospital - Rabindranath Tagore International Institute of Cardiac Sciences, Kolkata, IND

**Keywords:** cabg and cholecystectomy, valve repair and cholecystectomy, cholecystectomy and cardiac surgery, concomitant laparoscopic surgery, systematic review, coronary artery bypass grafting (cabg), laparoscopic cholecystectomy (lc), open heart surgery (ohs), cholecystitis and cardiac disease, gallstone and valvular dysfunction

## Abstract

Significant valvular or coronary artery disease may co-exist in patients presenting with symptomatic cholelithiasis. Isolated laparoscopic cholecystectomy in these cases is often associated with cardiac complications. Addressing the cardiac condition first may result in flaring up of cholecystitis during postoperative recovery and is associated with adverse outcomes. Open-heart surgery followed by laparoscopic cholecystectomy during a single operative setting is an option in these situations. The aim of our study is to review the published articles for this strategy and to share our initial experience with two such patients.

PubMed, OVID Medline, and Cochrane library database were used, and we searched these databases using Medical Subject Headings (MeSH) terms and keywords from the inception date until August 1, 2023, and did not restrict our search to any language, study type, sample size, or publication date. All the publications reporting concomitant laparoscopic cholecystectomy and open-heart surgery were identified and a systematic review was carried out.

Our first case underwent coronary artery bypass grafting and laparoscopic cholecystectomy. The second patient underwent a double valve replacement and laparoscopic cholecystectomy. Both the patients made an uneventful recovery, and are alive and doing well. Concomitant open-heart surgery and laparoscopic cholecystectomy in certain situations may be necessary and can be performed safely.

## Introduction and background

Abdominal surgeries in patients with ischemic or valvular heart disease carry a high risk of morbidity and mortality. The usual practice in this scenario is to correct the cardiac condition by coronary revascularization and repairing or replacing the affected cardiac valve before the abdominal operation is performed. Gallstone disease is a common disorder that may be concomitantly present in patients with cardiac disorders. While the true incidence of gallstones in the presence of coronary artery disease has not been established, the reported incidence of gastrointestinal complications is 4.1% in the presence of coronary artery disease [1]. While laparoscopic cholecystectomy (LC) is the preferred treatment option for symptomatic cholelithiasis, there remains a risk of developing myocardial infarction, heart failure, or arrhythmias in the presence of coronary artery disease and valvular heart disease, which may cause unwarranted morbidity and mortality. Therefore, elective LC is commonly performed after the cardiac condition is medically optimized or appropriate surgery is undertaken. However, acute cholecystitis (AC) may rarely develop in the postoperative period after the cardiac surgery, the incidence of which is 0.1 to 0.34% with an overall mortality of 23 to 45% as reported in various articles [1,2]. Although there are limited reports on the outcome of open-heart surgery (OHS) followed by LC in a single operative setting, this may be an option in such a situation. We have reviewed the published articles and shared our initial experience with two such cases where concomitant LC and OHS were performed with a good result.

## Review

Methods

Data Sources and Search Strategy

**Figure 1:**
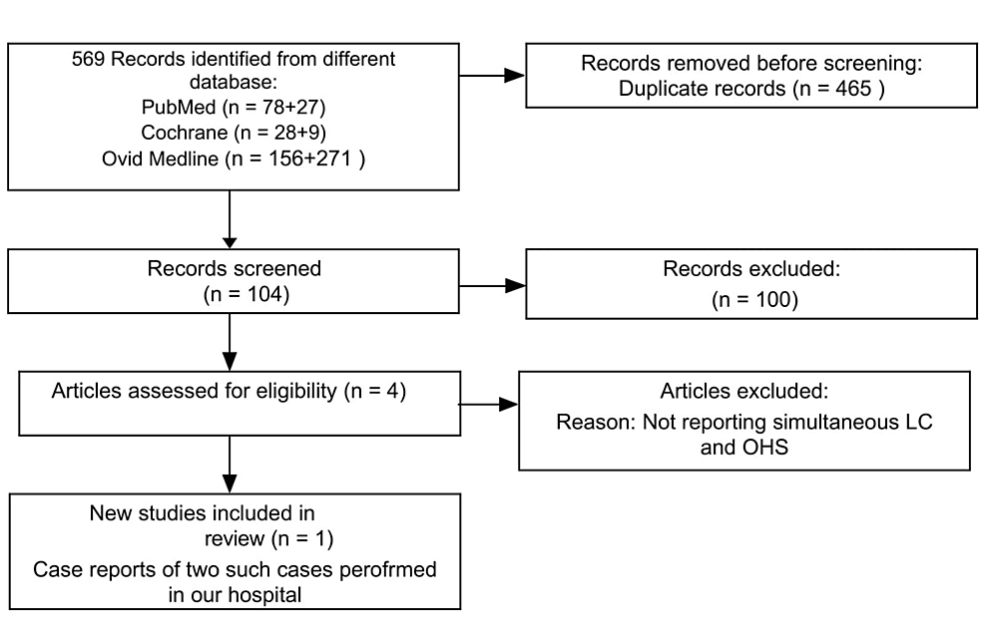
Flow Diagram for Search Strategy Using PRISMA Guidelines LC: laparoscopic cholecystectomy; OHS: open-heart surgery; PRISMA: preferred reporting items for systematic review and meta-analysis. The flow diagram was drawn by the authors of this article.

With our established search strategy, PubMed, OVID Medline, and Cochrane library database, we searched using Medical Subject Headings (MeSH) terms and keywords in two different combinations. The first one was “Laparoscopic Cholecystectomy” AND “Open Heart Surgery” and another was “Laparoscopic Cholecystectomy” AND “Coronary Artery Bypass Graft surgery”(CABG). We searched databases from the inception date until August 01, 2023, and did not restrict our search to any language, study type, sample size, or publication date. We used MeSH terms (“Laparoscopic Cholecystectomy AND Open Heart Surgery”, “Laparoscopic Cholecystectomy and Coronary Artery Bypass Graft”) for the search string. In addition, we scanned the reference lists of articles of eligible studies for further relevant articles. After excluding duplicate articles and studies unrelated to concomitant LC and OHS or CABG, selected articles were studied in detail. We followed Preferred Reporting Items for Systematic Reviews and Meta-Analysis (PRISMA) guidelines and prepared a flow diagram for the search strategy (Figure 1).

Data Extraction

One of the authors (SM) independently extracted all the selected articles into a preformatted spreadsheet and it was checked by another author (SKD). Both authors independently read the titles and abstracts of all the extracted articles. We then prepared a list of articles for detailed evaluation which had four articles with simultaneous LC and OHS or CABG surgery. We did not find any discrepancy in the final selected list of articles. Then, we analyzed these studies in detail. We decided not to carry out a meta-analysis as there was limited data with significant heterogeneity.

Study Details

We recorded the details for each study in Table 1.

**Table 1:** Details of the Studies Included in Systematic Review of Concomitant Laparoscopic Cholecystectomy and Open-Heart Surgery LC: laparoscopic cholecystectomy; OHS: open-heart surgery; N/A: not applicable; NA: not available; GSD: gallstone disease; TVD: triple vessel disease; MS: mitral valve stenosis; MI: myocardial infarction; AS: aortic valve stenosis; DVD: double vessel disease; SVD: single vessel disease; CABG: coronary artery bypass graft surgery; OC: open cholecystectomy; MVR: mitral valve replacement; AVR: aortic valve replacement; ERCP: endoscopic retrograde cholangiopancreatography; TVR: tricuspid valve repair

	Hekmat et al. [3]	Charokopos et al. [4]	Erdogan et al. [5]	Kahraman et al. [6]	Our Case Reports
Year of publication	2005	2007	2017	2019	NA
Study design	Case reports	Retrospective observational study	Retrospective observational study	Prospective observational study	Case reports
Journal	Journal of Cardiac Surgery	Surgery Today	The American Surgeon	The Heart Surgery Forum	NA
Number of surgeons	NA	NA	3	NA	02
Number of center	01	01	01	01	01
Number of participants	2	9	28	19	2
LC for symptomatic GSD	2	9	28	9	2
LC for asymptomatic GSD	0	0	0	2	0
OHS for TVD	1	4	NA	NA	1
OHS for DVD	NA	NA	NA	3	NA
OHS for SVD	NA	NA	NA	5	NA
OHS for severe MS	1	NA	NA	NA	NA
OHS for severe AS	NA	1	NA	NA	NA
OHS for severe MS and AS	NA	NA	NA	NA	1
OHS for previous MI	NA	4	NA	2	NA

We also recorded the indication and duration for LC and heart surgery (including the type of surgery). We performed two such cases with concomitant LC and OHS at our institution. We grouped the study outcomes in Table 2.

**Table 2:** Outcome of the Studies Included in Systematic Review of Concomitant Laparoscopic Cholecystectomy and Open-Heart Surgery. ICU: intensive care unit; CABG: coronary artery bypass graft surgery; LC: laparoscopic cholecystectomy; OHS: open-heart surgery; NA: not available

Author(s)	Hekmat et al. [3]	Charokopos et al. [4]	Erdogan et al. [5]	Kahraman et al. [6]	Our Case Reports
Year of publication	2005	2007	2017	2019	NA
Duration of LC (minutes)	NA	NA	42.2±13.1	56.5±15.6	52.5
Duration of OHS (minutes)	NA	NA	117.5±49.6	259±18.9	240
Duration of intubation (hours)	NA	25.7±6.7	11.6±6.2	17±9.6	18
Duration of ICU stay	6.5	4.1 ±1.6	3.1 ±1.4	3.1 ±1.4	3
Cholecystectomy related complications	None	None	4(14.3%)	None	None
CABG related complications	None	None	5(17.86%)	None	None
Pulmonary complications	NA	1(11.1%)	None	1(9.09%)	None
Duration of hospital stay (days)	6.5	19.2±5.7	16.5 ±6.3	14.2 ±3.7	8
In-hospital mortality	0(0%)	0(0%)	0(0%)	0(0%)	0(0%)
Readmission	NA	NA	0	NA	0
Need for re-intervention	NA	NA	3(10.71%)	NA	0
Mortality	NA	NA	1	NA	0

Results

LC along with OHS was carried out in two patients. OHS was done first in both cases. One of the patients was a 55-year-old non-smoker gentleman with known triple vessel disease who also developed symptomatic cholelithiasis. He was suffering from ischemic heart disease for 6 months and also had an episode of biliary colic in the interim. On ultrasound, he was diagnosed with cholelithiasis. After due deliberation, it was decided to perform coronary artery bypass and cholecystectomy as a single-stage procedure. A combined off-pump coronary artery bypass was performed followed by a standard four-port LC by the surgical laparoscopy team. The patient was extubated on the same day and kept under observation in the ICU for three days. His postoperative recovery was uneventful, and he was discharged on the seventh postoperative day. At the 36-month follow-up, he remains stable.

The second patient was a 40-year-old non-diabetic female who presented with AC. During a routine preoperative workup, she was found to have severe aortic as well as mitral valve disease. The valvular disease was severe, and she was felt to be a very high anesthetic risk. As a result, a decision to perform concomitant surgery was recommended in which aortic and mitral valve replacements were carried out through midline sternotomy using cardiopulmonary bypass with mild hypothermia and cardioplegic arrest, followed by LC. The patient was extubated on the same day. Intravenous heparin infusion was started the next day along with warfarin. Heparin was stopped once the international normalized ratio (INR) was 1.5. The patient made an uneventful recovery and was discharged on the seventh postoperative day. The patient remains well at the 36-month follow-up.

Both patients were very carefully evaluated by the “Heart Team,” which comprised a cardiologist, cardiac surgeon, and anesthetists along with the general surgery team. All relevant investigations and clinical details were assessed carefully. Following the discussion, the patient, as well as the family, were briefed about the different options, the risks, and the benefits, and with their consent the procedure was undertaken.

Discussion

Concomitant operations are surgical procedures that are performed on two or more anatomical sites for unrelated diseases. These are commonly performed in cases of trauma. Also, various general surgery and gynecological procedures are done in a single anesthetic setting which includes benign and malignant diseases. However, significant cardiac disease precludes elective surgery such as LC. In such cases, coronary revascularization for arterial occlusive disease such as CABG or percutaneous transluminal coronary angioplasty (PTCA) can reduce perioperative cardiovascular risks.

While PTCA offers a shorter period of recovery as compared to OHS, these patients require intensive antiplatelet therapy for 3-6 months and sometimes for 12 months before it can be interrupted. Thus, a non-cardiac surgery within the first 30 days following PTCA may cause hemorrhagic complications in about 9.5% of the patients with a mortality rate of 12% where bleeding is encountered [7]. Further, there is a risk of in-stent thrombosis even after 3 months following PTCA when the antiplatelet therapy is stopped temporarily for non-cardiac surgery. This may be the reason why some studies recommend that any elective surgery should be done 12 months after the placement of a drug-eluting coronary artery stent [8]. Similarly, the recovery is prolonged following OHS in patients undergoing CABG and cardiac valve replacement. Also, the patients with mechanical heart valves will be on anticoagulant medication. Stopping the anticoagulant drug may result in thromboembolic complications, which require bridging with heparin or low-molecular-weight heparin (LMWH) when elective non-cardiac surgery is performed, preferably 12 months after the cardiac surgery. Patients with symptomatic gallstone disease may also have a critical heart condition requiring intervention. Cholecystectomy without treating the coexisting cardiac ailment caries significantly increased morbidity and mortality. On the contrary, OHS patients may have gastrointestinal (GI) complications in the postoperative period. Although these complications are rare ranging from 0.5 to 2.1 %, AC is the cause in about 6-18% of the patients with a reported mortality of 15-42% in different studies [9-11].

AC following OHS may develop with or without gallstone and its management remains controversial. It has been reported that 78 patients developed GI complications in the postoperative period following 161576 open-heart operations done between January 1995 to December 2005 with an overall in-hospital mortality of 1.67%. Among these, 18 patients had AC and five of the patients had gallstones. While nine patients responded to conservative treatment within 48 hours, three patients with calculus cholecystitis (CC) and six with acalculous cholecystitis (ACC) underwent cholecystectomy. In this study, one patient with CC and two with ACC died following cholecystectomy with overall mortality of 28% [12]. In another report, the patient developed CC 20 days after CABG, where the right coronary artery received a bypass graft from the right gastroepiploic artery (RGEA). The patient was treated conservatively and LC was performed 9 months later, during which RGEA actually obstructed the operating field requiring another telescope to monitor and avoid injury to the RGEA vascular pedicle [13].

Therefore, performing LC and OHS as one-stage procedures in certain situations may be useful. While, the presence of anticoagulants or antiplatelet agents is not ideal prior to elective surgical procedures, after concomitant cardiac surgery all anticoagulants are actively reversed to achieve hemostasis and therefore bleeding complications have been seldom reported.

A series reporting concomitant surgeries in OHS reported that out of 1257 OHS performed between January 1999 and June 2004, only nine patients had concomitant cholecystectomy and two of them had concomitant LC without any mortality. However, the patients who had concomitant LC and OHS had longer ICU and hospital stays when compared with OHS patients alone (4.1SD1.6 and 19.2SD5.7 vs. 2.4SD1.2 and 9.1SD1.7) [4]. Similar successful outcomes have been reported by other papers, too [3]. A bigger series reported 28 patients undergoing concomitant LC and OHS, out of 2773 cardiac surgeries performed from January 2008 to September 2014. It is important to notice in the study that the LC was performed by the same surgeon who did OHS. Although there was no mortality, two patients developed LC-related serious complications. While one patient had a bile leak and the other one developed subhepatic collection, both of them were successfully treated with conservative management during the index hospitalization. A third patient had an umbilical trocar site hernia as a late complication which was electively repaired two years later [5]. In our series, the OHS was done by an experienced cardiac surgeon while LC was performed by a GI laparoscopic surgical team with a good outcome. Concomitant CABG and LC in 10 patients have been reported in another study. In the same study, nine patients underwent LC two or three days after PCI. There were no GI complications, bleeding, or mediastinitis in any of the 19 patients, suggesting the feasibility of the combined procedure, although the ICU and hospital stays were prolonged in the CABG group [6].

Our study has certain limitations that need highlighting. A small sample size comprising only two cases restricts the generalizability of findings. Additionally, the focus on a single-center experience raises concerns about the representativeness of outcomes across diverse settings. The potential for bias, including selection and reporting biases, may impact the study's objectivity and warrant a cautious interpretation of the reported results.

Future research should explore the efficacy and safety of concomitant LC and OHS through multicenter studies with long-term follow-up and comparative analyses. Additionally, investigations into risk stratification models and economic implications, as well as incorporating patient-reported outcomes, would enhance the understanding and applicability of this combined surgical approach.

## Conclusions

Concomitant LC and CABG surgery can be performed safely and can provide another option for the management of these conditions in certain situations when they co-exist. The operating team should include a cardiac surgeon as well as a surgeon experienced in LC for a satisfactory outcome.
